# Exploring pathophysiological insights to improve diagnostic utility of ultrasound markers for distinguishing placenta accreta spectrum from uterine‐scar dehiscence

**DOI:** 10.1002/uog.29144

**Published:** 2024-12-15

**Authors:** T. Adu‐Bredu, R. A. Aryananda, S. Mathewlynn, S. L. Collins

**Affiliations:** ^1^ Nuffield Department of Women's and Reproductive Health University of Oxford Oxford UK; ^2^ Anatomical Pathology Department, Dr Soetomo Academic General Hospital Universitas Airlangga Surabaya Indonesia

**Keywords:** abnormally invasive placenta, Cesarean section, hysterectomy, maternal morbidity, morbidly adherent placenta, ultrasound, uterine‐sparing surgery

## Abstract

**Objective:**

Accurate differentiation between placenta accreta spectrum (PAS) and uterine‐scar dehiscence with underlying non‐adherent placenta is often challenging, even for PAS experts, both prenatally and intraoperatively. We investigated the use of standardized two‐dimensional grayscale ultrasound and Doppler imaging markers in differentiating between these closely related, yet distinct, conditions.

**Methods:**

This was a retrospective cohort study conducted in two centers with specialized PAS services. All consecutive women with at least one previous Cesarean delivery and a current pregnancy with a low‐lying placenta or placenta previa, for whom detailed prenatal ultrasound, management and outcome information was available for review by the research team, were included. PAS was diagnosed clinically by the abnormal adherence of the placenta to the uterus. The PAS cases were classified using the International Federation of Gynecology and Obstetrics clinical classification. Grade 1 was considered low‐grade PAS while Grades 2 and 3 were classified as high‐grade PAS. The ultrasound markers were categorized according to their underlying pathophysiology, including lower uterine segment (LUS) remodeling, uteroplacental vascular remodeling and serosal hypervascularity. The combined ultrasound features were analyzed among the PAS and non‐PAS subgroups using the chi‐square test or Fisher's exact test, and univariable and multivariable logistic regression analysis. Additionally, receiver‐operating‐characteristics (ROC) curves were used to evaluate the diagnostic accuracy of the combined ultrasound features in differentiating between high‐grade PAS and uterine‐scar dehiscence.

**Results:**

Out of the 150 cases retrieved, six cases were excluded for not meeting the eligibility criteria. The included 144 cases comprised 89 cases of PAS, 23 cases of uterine‐scar dehiscence and 32 cases of uncomplicated low‐lying placenta or placenta previa. Among the PAS cases, there were 16 cases of low‐grade PAS and 73 of high‐grade PAS. Combined signs of LUS remodeling were present in most cases of uterine‐scar dehiscence (20/23 (87.0%)) and high‐grade PAS (67/73 (91.8%)) (*P* = 0.444), while these signs were absent in cases of low‐grade PAS (0/16) and uncomplicated low‐lying placenta or placenta previa (0/32). A subgroup analysis of cases with all LUS remodeling features present revealed that the combined signs of serosal hypervascularity (adjusted odds ratio (aOR), 41.2 (95% CI, 7.5–225.3)) and uteroplacental vascular remodeling (aOR, 116.0 (95% CI, 15.3–878.3)) were significantly associated with high‐grade PAS. Diagnostic accuracy testing within this subgroup revealed an area under the ROC curve (AUC) of 0.90 (95% CI, 0.81–0.99), sensitivity of 89.6% (95% CI, 79.7–95.7%) and specificity of 90.0% (95% CI, 68.3–98.8%) for the diagnosis of high‐grade PAS when all signs of uteroplacental vascular remodeling were present. If both signs of serosal hypervascularity were present, the AUC was 0.84 (95% CI, 0.74–0.95) with a sensitivity of 83.6% (95% CI, 72.5–91.5%) and specificity of 85.0% (95% CI, 62.1–96.8%) for the diagnosis of high‐grade PAS.

**Conclusions:**

The combined ultrasound markers of LUS remodeling are common in both high‐grade PAS and uterine‐scar dehiscence, while the combined features of abnormal vascularity (uteroplacental vascular remodeling and serosal hypervascularity) are specific to high‐grade PAS. Understanding these pathophysiological differences would enhance the diagnostic accuracy of ultrasound in distinguishing between these two conditions. © 2024 The Author(s). *Ultrasound in Obstetrics & Gynecology* published by John Wiley & Sons Ltd on behalf of International Society of Ultrasound in Obstetrics and Gynecology.

## INTRODUCTION

It is well‐established that the outcome of placenta accreta spectrum (PAS) is better when delivery occurs in a specialist center with appropriate preoperative planning^1^. While a definitive diagnosis of PAS can only be made intraoperatively when the placenta fails to separate from the uterus, in the hands of expert sonographers ultrasound has been proven to have excellent diagnostic accuracy^2^. An important condition that mimics high‐grade PAS both prenatally and intraoperatively, which often eludes experts in the field, is uterine‐scar dehiscence with an underlying non‐adherent placenta^3^. In these cases, the placenta separates from the uterus, surrounding the uterine defect, with minimal blood loss, enabling repair of the lower segment and conservation of the uterus. However, the confusion of uterine‐scar dehiscence with high‐grade PAS often leads to aggressive PAS‐appropriate management approaches^4^, which has been a subject of controversy in recent years. Some experts argue that uterine‐scar dehiscence is complicit in the pathophysiology of PAS^5^, ^6^, ^7^, while others believe that differentiating between these two conditions prenatally is unnecessary, as they both carry a risk of morbidity^8^, ^9^. We argue that, even though uterine‐scar dehiscence may result occasionally in a hysterectomy, particularly in larger defects close to the cervix^10^, ^11^, the morbidity risks of these two conditions are not comparable. High‐grade PAS is marked by extensive, friable hypervascularity, often involving the urinary bladder^12^, which increases the risk of massive hemorrhage and severe outcome. In contrast, uterine‐scar dehiscence occurs in a scarred uterus regardless of the location of the placenta^13^ and lacks this intricate abnormal hypervasculature. Therefore, accurate prenatal differentiation between these two conditions is crucial for comprehensive preoperative planning and resource allocation.

Currently, two‐dimensional (2D) grayscale and Doppler ultrasound serve as screening tools for patients at high risk for PAS, with ultrasound findings influencing preoperative preparation and management approaches^14^. In recent years, there has been an improved understanding of the underlying pathophysiology of PAS and how these morphological changes manifest on ultrasound^5^, ^15^, ^16^. This evolving understanding offers an opportunity to explore how these ultrasound signs could be utilized effectively in assessing cases with a high risk of PAS. In this study, we investigated the utility of the standardized 2D grayscale and Doppler imaging markers of PAS^17^, grouped according to their pathophysiological origin, in evaluating suspected PAS cases and differentiating them from uterine‐scar dehiscence with an underlying non‐adherent placenta.

## METHODS

This was a retrospective cohort study carried out in two hospitals with PAS specialist centers. Data were obtained from the John Radcliffe Hospital, Oxford, UK, between January 2019 and December 2022, and the Dr Soetomo Academic General Hospital, Surabaya, Indonesia, between July 2022 and October 2023. Ethical approval was obtained from the institutional review boards with reference numbers 14/NS/0069 and 1169/LOE/301.4.2/XII/2022, respectively, prior to the collection of data.

### Eligibility criteria

Data on all cases of ongoing pregnancy with placenta previa or a low‐lying placenta who had at least one previous Cesarean delivery during the specified period were retrieved and the prospectively acquired data were reviewed. Cases were excluded if they lacked detailed ultrasound findings reported using the standard imaging descriptors of PAS^17^, with no good quality images and videos to complement the findings. PAS cases were also excluded if they lacked a clearly defined intraoperative diagnosis and classification based on the International Federation of Gynecology and Obstetrics (FIGO) classification^18^.

### Diagnostic criteria and clinical grading of PAS

Placenta previa was defined as the presence of the placenta overlying the internal cervical os. A low‐lying placenta was defined when the lower edge of the placenta was less than 20 mm from the internal cervical os. Uncomplicated placenta previa or low‐lying placenta (hereafter referred to as uncomplicated placenta previa) was diagnosed intraoperatively by the presence of a normal‐looking lower uterine segment (LUS) and spontaneous placental separation.

**Figure 1:**
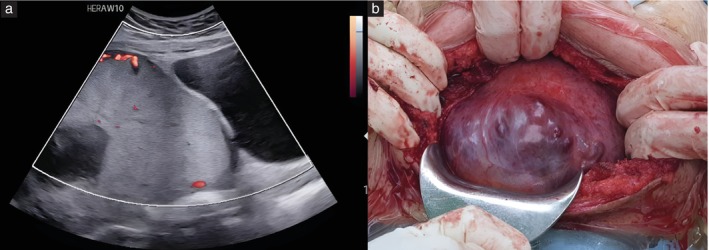
(a) Color Doppler ultrasound image in a patient with two previous Cesarean deliveries and anterior placenta previa with ultrasound features of lower uterine segment remodeling (placental bulge, myometrial thinning, loss of clear zone) and absence of uteroplacental vascular remodeling and serosal hypervascularity. (b) The intraoperative image demonstrates a wide area of uterine‐scar dehiscence, with the placenta visualized directly underneath the defect. Note absence of hypervascularity on the serosal surface. The placenta separated spontaneously from the uterine wall, confirming the diagnosis of uterine‐scar dehiscence with an underlying non‐adherent placenta.

**Figure 2:**
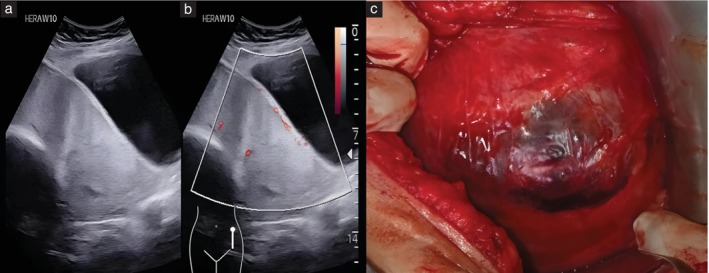
(a,b) Ultrasound images in a patient with two previous Cesarean deliveries and anterior placenta previa. (a) On grayscale ultrasound, there was significant myometrial thinning at the lower uterine segment, with loss of the retroplacental clear zone. (b) Color Doppler ultrasound revealed the absence of uteroplacental vascular remodeling and serosal hypervascularity features. (c) The intraoperative image demonstrates an obvious placental bulge, with the placenta visible underneath the scar and absence of hypervascularity on the serosal surface. The placenta separated spontaneously from the uterine wall, confirming the diagnosis of uterine‐scar dehiscence with an underlying non‐adherent placenta.

Cases were classified as uterine‐scar dehiscence intraoperatively in the presence of a thinned, transparent LUS with an obvious placental bulge and the noticeable absence of hypervascularity on the serosal surface, with complete placental separation from the uterus surrounding the area of compromised myometrium (Figures 1 and 2).

**Figure 3:**
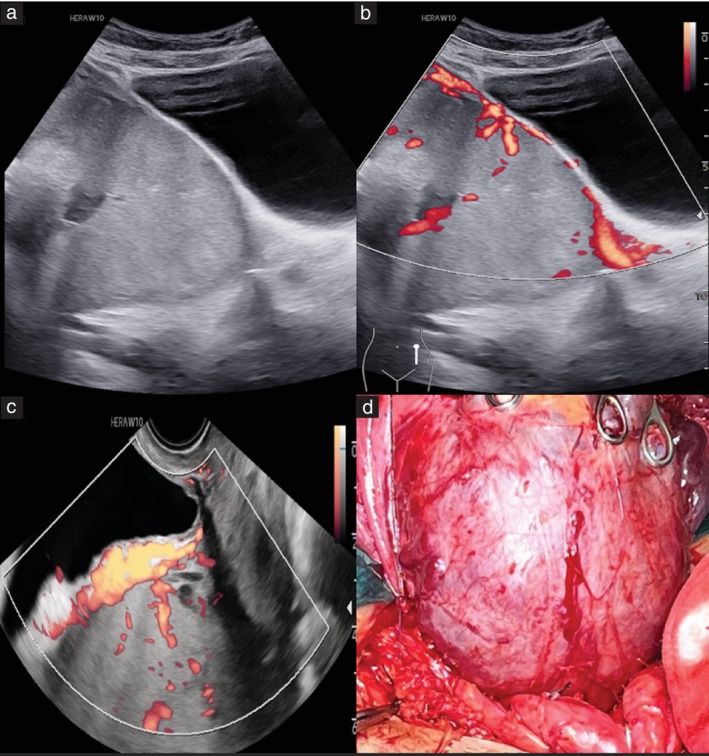
Ultrasound images in a patient with one previous Cesarean delivery and anterior placenta previa. (a) Grayscale ultrasound image showed loss of retroplacental clear zone, absent lacunae and absent bladder wall interruption. Transabdominal (b) and transvaginal (c) color Doppler images showed subplacental hypervascularity. All other abnormal color Doppler features, such as lacunae and serosal hypervascularity, were noticeably absent. (d) The intraoperative image shows an apparently normal lower uterine segment. A diagnosis of low‐grade placenta accreta spectrum was made based on the inability to separate the placenta from the uterine wall, as it remained ‘glued’ to the uterus.

**Figure 4:**
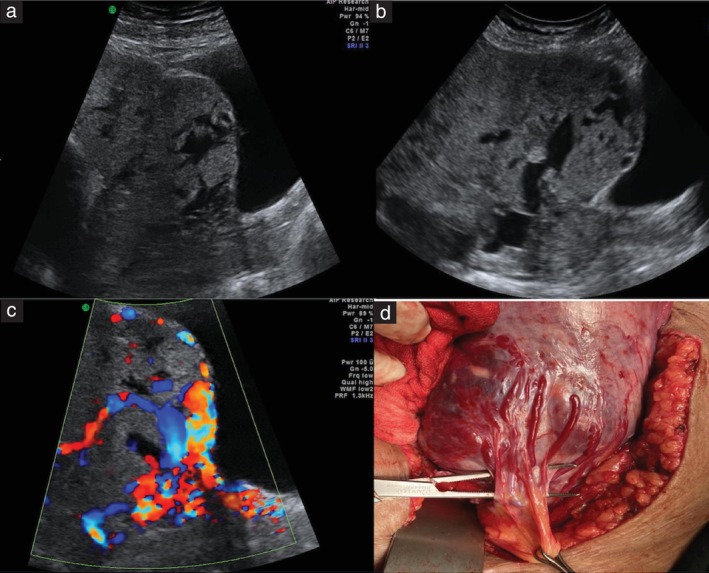
(a,b) Grayscale ultrasound images in a patient with two previous Cesarean deliveries and anterior placenta previa with features of extensive lower uterine segment remodeling (placental bulge, myometrial thinning and loss of clear zone). (c) On color Doppler imaging, features of uteroplacental vascular remodeling (abnormal lacunae, lacunae feeding vessel and uterovesical hypervascularity) as well as serosal hypervascularity (bridging vessels and bladder wall interruption) were observed. (d) The intraoperative image shows a placental bulge and numerous vessels on the serosal surface, with anastomosis to the vesical vessels. A diagnosis of high‐grade placenta accreta spectrum was made based on the intraoperative appearance and the failure of placental separation.

**Figure 5:**
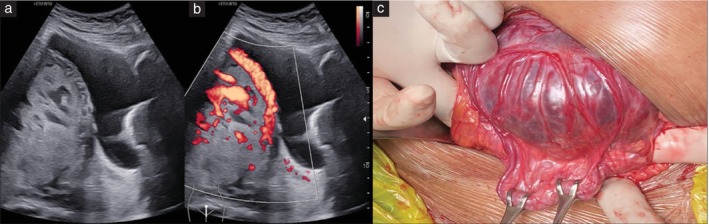
(a) Grayscale ultrasound image in a patient with two previous Cesarean deliveries and anterior placenta previa, showing signs of extensive lower uterine segment remodeling (placental bulge, myometrial thinning and loss of clear zone). (b) On color Doppler imaging, features of uteroplacental vascular remodeling (subplacental hypervascularity, lacunae and lacunae feeding vessels) and serosal hypervascularity (bridging vessels and bladder wall interruption) were observed. (c) The intraoperative image shows a thinned lower uterine segment with a placental bulge, with massive vascularity on the serosal surface running craniocaudally, with anastomosis to the vesical vessels. A diagnosis of high‐grade placenta accreta spectrum was made based on the intraoperative appearance and the failure of placental separation.

PAS was diagnosed clinically when the placenta appeared to be ‘glued’ to the uterine wall in the hysterectomy specimen or from the focally excised specimen. The intraoperative appearance of the PAS cases was classified according to the FIGO clinical classification from findings at laparotomy^18^. For the purpose of this study, PAS was classified as low‐grade or high‐grade. Low‐grade PAS (FIGO Grade 1) was defined as the presence of a normal‐appearing LUS with minimal or no hypervascularity on the serosal surface, a positive ‘dimple sign’ and failure of placental separation (Figure 3). High‐grade PAS (FIGO Grades 2 and 3) was defined as the presence of an obvious placental bulge, with or without involvement of nearby visceral structures and hypervascularity seen on the serosal surface (Figures 4 and 5).

Histological diagnosis was based on the FIGO classification^18^. The diagnosis of PAS was established by the absence of intervening decidua between the placental villi and the myometrium. Placenta accreta was defined as the attachment of the placental villi to the myometrium, and placenta increta was defined as the presence of placental villi within the myometrial layer. The diagnosis of placenta percreta was made by the presence of placental villi within, or that breeched, the uterine serosa.

### Management approach

The management approach for PAS differed between the two centers. At John Radcliffe Hospital, PAS was managed by Cesarean hysterectomy or intentional placental retention. At Dr Soetomo Academic General Hospital, the intraoperative topographic classification of PAS guided the choice of management, whether by Cesarean hysterectomy, modified subtotal hysterectomy or uterine‐sparing surgery^19^. Cases of uncomplicated placenta previa or uterine‐scar dehiscence with non‐adherent placenta were managed with uterine‐sparing surgery.

### Analysis of ultrasound signs

Ultrasound assessment was performed by S.L.C. and R.A.A., who both possess extensive experience and expertise in PAS. Both transabdominal and transvaginal ultrasound approaches were used complementary to each other, and assessment was performed with a full urinary bladder. Results were reported using the standardized imaging descriptors of PAS^17^. On retrieval of the ultrasound data, all ultrasound images were reviewed by a second operator (T.A‐.B.) with experience and expertise in PAS ultrasound. Any discrepancies observed were discussed with R.A.A. and S.L.C.

The standardized 2D imaging descriptors, namely loss of the clear zone, abnormal placental lacunae, bladder wall interruption, myometrial thinning, placental bulge, uterovesical hypervascularity, subplacental hypervascularity, bridging vessels and placental lacunae feeding vessels^17^, were reviewed by the authors. These ultrasound signs were classified based on their known pathophysiological correlation, specifically focusing on LUS remodeling, uteroplacental vascular remodeling and serosal hypervascularity. For instance, placental bulge occurs in the presence of myometrial thinning and is a manifestation of extensive LUS remodeling. The presence of bridging vessels on color Doppler ultrasound and bladder wall interruption on 2D grayscale ultrasound represents the presence of serosal hypervascularity. The presence of abnormal placental lacunae, lacunae feeding vessels, uterovesical and/or subplacental hypervascularity is a manifestation of extensive remodeling of the uteroplacental vasculature with distortion of the placental architecture. These collective ultrasound features correlate with both intraoperative and histological findings. A detailed description of the pathophysiology and the related ultrasound signs of PAS is presented in Table 1.

**Table 1:** Ultrasound signs generated by underlying pathophysiology in placenta accreta spectrum (PAS)

Key features	Ultrasound signs	Pathophysiology
Lower uterine segment remodeling	Myometrial thinning, placental bulge, loss of retroplacental clear zone	Defective myometrial healing results in fibrosis and reduced smooth muscle density at area of scar. As a result, uterine architecture is distorted, manifesting as progressive myometrial thinning as the pregnancy advances, which is then accompanied by placental bulge^16^. On 2D grayscale ultrasound, the retroplacental clear zone is not observed but it can be seen on advanced 3D volume rendering ultrasound with contrast enhancement technique^20^
Uteroplacental vascular remodeling	Abnormal placental lacunae, lacunae feeding vessels, uterovesical and/or subplacental hypervascularity	Deep penetration of extravillous trophoblast results in abnormal dilatation of deep myometrial arteries (radial and arcuate), manifesting as hypervascular areas behind placenta^15^, ^21^. Lacunae result from distortion of placental cotyledons owing to high‐velocity blood flow entering intervillous spaces from remodelled myometrial arteries^22^
Serosal hypervascularity	Bladder wall interruption, bridging vessels	Extensive network of large new blood vessels seen on serosa overlying area of PAS. Walls of these vessels have immature architecture with disorganized muscular layer making them very friable. This hypervascularity appears to result from down regulation of Von Willebrand factor expression^23^

2D, two‐dimensional; 3D, three‐dimensional.

### Statistical analysis

Data were collated using Microsoft Excel (Microsoft Cooperation, Redmond, WA, USA) and exported to IBM SPSS statistical software version 29 (IBM, Chicago, IL, USA) and R software version 4.3.1 (R Foundation, Vienna, Austria)^24^ for statistical analysis. Categorical variables are described as *n* (%) and were compared using the chi‐square test or Fisher's exact test. The Shapiro–Wilk normality test^25^ was performed on numerical variables to determine the distribution of the data, which in turn influenced the choice between the parametric or non‐parametric statistical tests used. Univariable and multivariable logistic regression were used to determine the association between the ultrasound features and the clinical diagnosis. The diagnostic accuracy of the ultrasound parameters in diagnosing high‐grade PAS was determined using receiver‐operating‐characteristics (ROC) curves. Statistical significance was set at *P* < 0.05.

## RESULTS

### Description of cohort

Data on a total of 150 cases were retrieved from the two centers. Six cases were excluded as they did not meet the eligibility criteria; therefore, 144 cases were analyzed, comprising 89 cases of PAS and 55 cases with uncomplicated uterine‐scar dehiscence or uncomplicated low‐lying placenta or placenta previa. The PAS cases comprised 16 cases of low‐grade PAS and 73 cases of high‐grade PAS. Histologically, out of the total number of PAS cases, 16 (18.0%) cases of placenta accreta, 60 (67.4%) cases of placenta increta and 11 (12.4%) cases of placenta percreta were diagnosed. Three cases were managed by intentional placenta retention, two of which were successful while the other case required an emergency hysterectomy owing to postpartum hemorrhage. The non‐PAS cases consisted of 23 cases of uterine‐scar dehiscence and 32 cases of uncomplicated low‐lying placenta or placenta previa. Demographic characteristics and management outcomes are presented in Table 2 and Table S1.

**Table 2:** Patient characteristics, management approach, surgical outcome and histological classification of placenta accreta spectrum (PAS), in cases with at least one previous Cesarean delivery (CD) and current pregnancy with low‐lying placenta or placenta previa, according to diagnosis of PAS

Characteristic	PAS (*n* = 89)	No PAS (*n* = 55)	*P*
Baseline characteristics			
Maternal age (years)	33.98 ± 4.17	33.4 ± 4.65	0.45¶
Parity			0.29§
1	32 (36.0)	25 (45.5)	
≥ 2	57 (64.0)	30 (54.5)	
Number of previous CD			0.44§
1	44 (49.4)	33 (60.0)	
2	35 (39.3)	18 (32.7)	
≥ 3	10 (11.2)	4 (7.3)	
Number of previous STOP			0.025§
0	58 (65.2)	47 (85.5)	
1	23 (25.8)	5 (9.1)	
≥ 2	8 (9.0)	3 (5.5)	
Gestational age at ultrasound (weeks)	33.53 ± 2.8	34.3 ± 2.25	0.038¶
Gestational age at delivery (weeks)	35.1 ± 2.47	36.85 ± 1.45	< 0.001¶
Management approach			
Uterine‐sparing surgery	41 (46.1)	55 (100)	< 0.001§
Cesarean hysterectomy	45 (50.6)	0 (0)	< 0.001§
Intentional placental retention	3 (3.4)†	0 (0)	0.283§
Outcomes			
Blood loss (mL)	1700 (1100–3200)	1600 (1000–3075)	< 0.001‡
Composite maternal morbidity*	15 (16.9)	0 (0)	< 0.001§
Histological classification of PAS			
Accreta	16 (18.0)	—	—
Increta	60 (67.4)	—	—
Percreta	11 (12.4)	—	—
Histology unavailable	2 (2.2)	—	—

Data are given as mean ± SD, *n* (%) or median (interquartile range).

* Composite maternal morbidity comprised visceral organ injury (bladder injury and bowel injury), massive postpartum hemorrhage, cardiomyopathy and spontaneous uterine rupture.

† Only two cases were successful.

‡ Mann–Whitney *U*‐test.

§ Chi‐square test or Fisher's exact test.

¶ Independent *t*‐test.

STOP, surgical termination of pregnancy.

The combined features of LUS remodeling (loss of clear zone, myometrial thickness < 1 mm and placental bulge) were present in 87 cases, including 67/73 (91.8%) cases with high‐grade PAS and 20/23 (87.0%) cases with uterine‐scar dehiscence (*P* = 0.444). These three combined features of LUS remodeling were not present in cases of low‐grade PAS (0/16) and uncomplicated low‐lying placenta or placenta previa (0/32) (Table 3). A subgroup analysis among cases with all three LUS remodeling features revealed that uteroplacental vascular remodeling (adjusted odds ratio (aOR), 116.0 (95% CI, 15.3–878.3)) and serosal hypervascularity (aOR, 41.2 (95% CI, 7.5–225.3)) were significantly associated with high‐grade PAS. Diagnostic accuracy testing within this subgroup for the prediction of high‐grade PAS revealed an area under the ROC curve (AUC) of 0.90 (95% CI, 0.81–0.99), a sensitivity of 89.6% (95% CI, 79.7–95.7%) and a specificity of 90.0% (95% CI, 68.3–98.8%) for the combined signs of uteroplacental vascular remodeling. If both signs of serosal hypervascularity were present, the AUC was 0.84 (95% CI, 0.74–0.95) with a sensitivity of 83.6% (95% CI, 72.5–91.5%) and a specificity of 85.0% (95% CI, 62.1–96.8%) for the diagnosis of high‐grade PAS (Table 4).

**Table 3:** Frequency of ultrasound signs according to intrapartum diagnosis of placenta accreta spectrum (PAS), uterine‐scar dehiscence or uncomplicated low‐lying placenta or placenta previa

Ultrasound signs	High‐grade PAS (*n* = 73)	Low‐grade PAS (*n* = 16)	Uterine‐scar dehiscence (*n* = 23)	Uncomplicated placenta previa (*n* = 32)	*P* *	*P* †	*P* ‡
Lower uterine segment remodeling							
Loss of clear zone	72 (98.6)	10 (62.5)	22 (95.7)	0 (0)	< 0.001	0.424	< 0.001
Myometrial thickness < 1 mm	73 (100)	10 (62.5)	23 (100)	0 (0)	< 0.001	1.0	< 0.001
Placental bulge	67 (91.8)	0 (0)	20 (87.0)	0 (0)	< 0.001	0.444	< 0.001
All three signs present	67 (91.8)	0 (0)	20 (87.0)	0 (0)	< 0.001	0.444	< 0.001
Uteroplacental vascular remodeling							
Abnormal placental lacunae	64 (87.7)	8 (50.0)	7 (30.4)	2 (6.3)	0.002	< 0.001	< 0.001
Lacunae feeding vessel	65 (89.0)	1 (6.3)	4 (17.4)	0 (0)	< 0.001	< 0.001	< 0.001
Subplacental hypervascularity	66 (90.4)	14 (87.5)	5 (21.7)	0 (0)	0.662	< 0.001	< 0.001
Uterovesical hypervascularity	69 (94.5)	5 (31.3)	5 (21.7)	1 (3.1)	< 0.001	< 0.001	< 0.001
All three signs present§	64 (87.7)	1 (6.3)	2 (8.7)	0 (0)	< 0.001	< 0.001	< 0.001
Serosal hypervascularity							
Bladder wall interruption	63 (86.3)	2 (12.5)	5 (21.7)	0 (0)	< 0.001	< 0.001	< 0.001
Bridging vessels	64 (87.7)	10 (62.5)	3 (13.0)	0 (0)	0.025	< 0.001	< 0.001
Both signs present	58 (79.5)	2 (12.5)	3 (13.0)	0 (0)	< 0.001	< 0.001	< 0.001

Data are given as *n* (%).

*P*‐values are for comparisons between:

* high‐grade PAS *vs* low‐grade PAS,

† high‐grade PAS *vs* uterine‐scar dehiscence and

‡ high‐grade PAS *vs* uncomplicated placenta previa.

§ Abnormal placental lacunae and lacunae feeding vessels and hypervascularity (subplacental and/or uterovesical).

**Table 4:** Association of ultrasound signs of uteroplacental vascular remodeling and serosal hypervascularity with presence of high‐grade placenta accreta spectrum (PAS) in women with all three signs of lower uterine segment remodeling present*

Feature	High‐grade PAS (*n* = 67)	Uterine‐ scar dehiscence (*n* = 20)	OR (95% CI)	Adjusted OR (95% CI)	AUC (95% CI)	Sensitivity (95% CI) (%)	Specificity (95% CI) (%)
Uteroplacental vascular remodeling†	60 (89.6)	2 (10.0)	77.1 (14.7–404.7)	116.0 (15.3–878.3)	0.90 (0.81–0.99)	89.6 (79.7–95.7)	90.0 (68.3–98.8)
Serosal hypervascularity‡	56 (83.6)	3 (15.0)	28.8 (7.2–115.5)	41.2 (7.5–225.3)	0.84 (0.74–0.95)	83.6 (72.5–91.5)	85.0 (62.1–96.8)

Data are given as *n* (%), unless stated otherwise.

Odds ratio (OR) adjusted for gestational age at ultrasound.

* Loss of clear zone, myometrial thickness < 1 mm and placental bulge.

† Abnormal placental lacunae, lacunae feeding vessel and hypervascularity (subplacental and/or uterovesical).

‡ Bridging vessels and bladder wall interruption.

AUC, area under the receiver‐operating‐characteristics curve.

All three combined features of LUS remodeling or uteroplacental vascular remodeling or both signs of serosal hypervascularity were not seen in any case of uncomplicated placenta previa. Also, most cases of low‐grade PAS did not present with these combined features (Table 3). Nevertheless, when considering the individual signs for PAS, the following were noted in more than 60% of low‐grade PAS cases: loss of the retroplacental clear zone, myometrial thinning, subplacental hypervascularity and bridging vessels (Table 3).

## DISCUSSION

The combined ultrasound features of LUS remodeling were observed in both high‐grade PAS and uterine‐scar dehiscence with an underlying non‐adherent placenta. However, combined features of abnormal vascularity (uteroplacental vascular remodeling and serosal hypervascularity) were associated specifically with high‐grade PAS but not uterine‐scar dehiscence. Diagnostic accuracy testing revealed an excellent specificity of 90% (95% CI, 68.3–98.8%) for uteroplacental vascular remodeling and a specificity of 85% (95% CI, 62.1–96.8%) for serosal hypervascularity in ruling out scar dehiscence with non‐adherent placenta. Although most low‐grade PAS cases do not present with combined ultrasound features of abnormal vascularity and rarely exhibit combined features of LUS remodeling, individual imaging features may be present. These features could be useful in distinguishing low‐grade PAS from uncomplicated placenta previa.

### Clinical implications

Full‐thickness uterine injury from previous Cesarean delivery leads to several alterations in the uterine healing pattern, which may result in elastosis, tissue edema and myofiber disarray^26^ with reduced smooth muscle density^27^. This altered healing process results in extensive architectural remodeling of the LUS that is complicit in the pathophysiology of PAS and uterine‐scar dehiscence^16^. In this study, we observed that the combined ultrasound features of LUS remodeling were noted in both uterine‐scar dehiscence with underlying non‐adherent placenta and high‐grade PAS, but not in low‐grade PAS or uncomplicated low‐lying placenta/placenta previa. These observations are consistent with intraoperative findings, in which uterine‐scar dehiscence and high‐grade PAS typically present with an obvious placental bulge and a thin LUS. In contrast, such extensive changes are not typically seen in cases of low‐grade PAS and uncomplicated placenta previa. With the presence of the retroplacental clear zone being the only direct marker of placenta separation currently known^28^, ^29^, ^30^, its visualization on 2D ultrasound imaging is often influenced by various technical factors, including operator‐dependent image optimization techniques, probe compression and resolution of the ultrasound equipment, which may impact on its specificity. Consequently, despite the presence of a non‐adherent placenta underneath the uterine‐scar dehiscence, the clear zone was not observed in 22 out of 23 cases (Table 3), which is consistent with the findings of other studies^3^, ^4^, ^10^, ^16^, ^31^. In a recent study, we demonstrated the efficacy of three‐dimensional advanced volume rendering and contrast enhancement techniques for assessment of the uteroplacental interface^20^. These techniques overcame the limitations of 2D imaging, and revealed the retroplacental clear zone in 13 out of 14 cases of uterine‐scar dehiscence, indicating the presence of a non‐adherent placenta underneath the uterine scar^20^.

The deep implantation of extravillous trophoblast results in the recruitment and extensive remodeling of the deep myometrial arteries (radial and arcuate) to enable placental growth and development^15^. This results in the anatomical distortion of the placental cotyledon due to high‐velocity feeding blood flow from the deep myometrial arteries and extensive dilatation of the local uteroplacental vessels^22^, ^32^, ^33^. Uteroplacental vascular remodeling manifests on ultrasound as abnormal lacunae, feeding vessels, subplacental hypervascularity and/or uterovesical hypervascularity^22^, ^34^. In addition to this, extensive hypervascularity is seen on the serosal surface and within the peritoneum^12^, possibly owing to downregulation of Von Willebrand factor expression^23^. This hypervascularity is seen as bridging vessels and bladder wall interruption on ultrasound^35^. In the present study, ultrasound markers for uteroplacental vascular remodeling and serosal hypervascularity were observed mostly in high‐grade PAS cases. In contrast, these abnormal vascular features were not observed in most cases of low‐grade PAS and uterine‐scar dehiscence. Similarly, our recent study revealed the inability of robustly created scoring systems to differentiate low‐grade PAS from uterine‐scar dehiscence^36^. Since PAS is not a binary condition, we hypothesize that the degree of trophoblastic invasion influences the extent of vascular remodeling, which may explain the low incidence of these combined features in low‐grade PAS. As cases of uterine‐scar dehiscence have normal placental attachment, there is understandably an absence of abnormal vascular features in most cases despite extensive LUS remodeling due to the formation of scar tissue.

While there were cases of high‐grade PAS without combined signs of abnormal vascularity, these may be explained by the concurrent presence of uterine‐scar dehiscence and PAS in the same placental bed. In such cases, the abnormal villi implantation may only be focal and superficial, but the progressive dehiscence of myometrial tissue behind and around the accreta area may contribute to the overt uterine remodeling changes observed intraoperatively, thus influencing the FIGO clinical classification.

### Strengths and limitations

The strength of this study lies in its novel approach of classifying standardized ultrasound markers of PAS according to their underlying pathophysiology. This classification approach and analysis provide valuable insights, guiding clinicians in differentiating high‐grade PAS from uterine‐scar dehiscence and enhancing the overall understanding of these ultrasound markers. A limitation of the study is its retrospective design. It is widely recognized that the definitive diagnosis of PAS or uterine‐scar dehiscence currently relies on the findings at delivery. Prior to this study, the diagnostic accuracy of these ultrasound features in identifying these conditions had not been explored. To address this, a retrospective study design was adopted, drawing on data obtained from specialist centers and employing a rigorous review of all cases. While we acknowledge that this approach potentially increases the risk of eliminating relevant cases, it ensured the accuracy of all data included in the analysis, thereby bolstering confidence in the results. Additionally, because the ultrasound images were reviewed and analysis finalized among the authors, our methodology did not permit inter‐rater reliability testing of the sonographic signs. Moving forward, a prospective multicenter cohort study using these findings could help to enhance our understanding of the diagnostic capabilities of grayscale and color Doppler ultrasound in distinguishing between PAS and uterine‐scar dehiscence with an underlying non‐adherent placenta.

### Conclusions

Both high‐grade PAS and uterine‐scar dehiscence with underlying non‐adherent placenta exhibit extensive remodeling of the LUS, leading to the distorted uterine architecture observed on 2D grayscale ultrasound. Nevertheless, the deep villous implantation that results in uteroplacental vascular remodeling and serosal hypervascularity is distinctive to high‐grade PAS. Using these ultrasound features could be valuable for distinguishing PAS from uterine‐scar dehiscence with an underlying non‐adherent placenta.

## Supporting information


**Table S1** Patient characteristics, management approach, surgical outcome and histological classification of placenta accreta spectrum (PAS), in cases with high‐grade PAS and those with uterine‐scar dehiscence

## Data Availability

The data that support the findings of this study are available from the corresponding author upon reasonable request.
