# Supplementary material for: Exploring pathophysiological insights to improve diagnostic utility of ultrasound markers for distinguishing placenta accreta spectrum from uterine‐scar dehiscence
Source: Ultrasound Obstet Gynecol. 2024 Dec 15;65(1):85–93. doi: 10.1002/uog.29144 (PMC11693827; doi:10.1002/uog.29144)
Supplement: Supplementary file 1 — Table S1 Patient characteristics, management approach, surgical outcome and histological classification of placenta accreta spectrum (PAS), in cases with high‐grade PAS and those with uterine‐scar dehiscence [file UOG-65-85-s001.docx]

| **Characteristic** | | **High PAS cases**  N = 73 | **Scar dehiscence cases**  N = 23 | **P value** |
| --- | --- | --- | --- | --- |
| ***Baseline characteristics*** | | | | |
| *Maternal age (years) | | 34.49 + 4.13 | 32.96 + 5.04 | 0.097 |
| **Parity | 1 | 23 (31.5%) | 4 (17.4%) | 0.29 |
|  | > 2 | 50 (68.5%) | 19 (82.6%) |  |
| **Number of previous CD | 1 | 35 (47.9%) | 11 (47.8%) | 0.51 |
|  | 2 | 29 (39.7%) | 11 (47.8%) |  |
|  | > 3 | 9 (12.3%) | 1 (4.3%) |  |
| **Number of previous STOPs | None | 45 (61.6%) | 19 (82.6%) | 0.176 |
|  | 1 | 20 (27.4%) | 3 (13.0%) |  |
|  | > 2 | 8 (10.96%) | 1 (4.3%) |  |
| ***Gestational age at ultrasound (weeks) | | 33.2 + 2.8 | 34.96 + 1.69 | 0.006 |
| ***Gestational age at delivery (weeks) | | 34.9 + 2.387 | 36.13 + 1.66 | < 0.001 |
| ***Management approach*** | | | | |
| ** Uterine sparing surgery | | 25 (43.2%) | 23 (100%) | < 0.001 |
| ** Caesarean hysterectomy | | 45 (61.6%) | 0 | < 0.001 |
| **Intentional placental retention | | 3 (4.1%) | 0 | 1.0 |
| ***Outcomes*** | |  | | |
| *Blood loss (ml) | | 2721.3 + 1813.3 | 1256.5 + 700.54 | < 0.001 |
| **Composite maternal morbidity | | 15 (20.5%) | 0 | 0.018 |
| ***Histological classification*** | | | | |
| **Accreta | | 13 (17.8%) | N/A | N/A |
| **Increta | | 49 (67.1%) | N/A | N/A |
| **Percreta | | 10 (13.7%) | N/A | N/A |
| **Histology unavailable | | 2 (2.7%) | N/A | N/A |
